# Combined LC-MS/MS feature grouping, statistical prioritization, and interactive networking in msFeaST

**DOI:** 10.1093/bioinformatics/btae584

**Published:** 2024-09-30

**Authors:** Kevin Mildau, Christoph Büschl, Jürgen Zanghellini, Justin J J van der Hooft

**Affiliations:** Bioinformatics Group, Department of Plant Sciences, Wageningen University & Research, Radix Building, Droevendaalsesteeg 1, Wageningen, 6708PB, the Netherlands; Department of Analytical Chemistry, University of Vienna, Vienna 1090, Austria; Doctoral School in Chemistry (DOSCHEM), University of Vienna, Vienna 1090, Austria; Department of Agrobiotechnology, Institute of Bioanalytics and Agro-Metabolomics, University of Natural Resources and Life Sciences, Konrad-Lorenz-Straße, Lower Austria 3430, Austria; Department of Analytical Chemistry, University of Vienna, Vienna 1090, Austria; Bioinformatics Group, Department of Plant Sciences, Wageningen University & Research, Radix Building, Droevendaalsesteeg 1, Wageningen, 6708PB, the Netherlands; Department of Biochemistry, University of Johannesburg, Johannesburg, Gauteng Province 2006, South Africa

## Abstract

**Summary:**

Computational metabolomics workflows have revolutionized the untargeted metabolomics field. However, the organization and prioritization of metabolite features remains a laborious process. Organizing metabolomics data is often done through mass fragmentation-based spectral similarity grouping, resulting in feature sets that also represent an intuitive and scientifically meaningful first stage of analysis in untargeted metabolomics. Exploiting such feature sets, feature-set testing has emerged as an approach that is widely used in genomics and targeted metabolomics pathway enrichment analyses. It allows for formally combining groupings with statistical testing into more meaningful pathway enrichment conclusions. Here, we present msFeaST (mass spectral Feature Set Testing), a feature-set testing and visualization workflow for LC-MS/MS untargeted metabolomics data. Feature-set testing involves statistically assessing differential abundance patterns for groups of features across experimental conditions. We developed msFeaST to make use of spectral similarity-based feature groupings generated using k-medoids clustering, where the resulting clusters serve as a proxy for grouping structurally similar features with potential biosynthesis pathway relationships. Spectral clustering done in this way allows for feature group-wise statistical testing using the globaltest package, which provides high power to detect small concordant effects via joint modeling and reduced multiplicity adjustment penalties. Hence, msFeaST provides interactive integration of the semi-quantitative experimental information with mass-spectral structural similarity information, enhancing the prioritization of features and feature sets during exploratory data analysis.

**Availability and implementation:**

The msFeaST workflow is freely available through https://github.com/kevinmildau/msFeaST and built to work on MacOS and Linux systems.

## 1 Introduction

Untargeted metabolomics deals with the comprehensive characterization of the composition of small chemicals, or metabolites, in biological samples. Typically, high-resolution Liquid Chromatography Tandem Mass Spectrometry (LC-MS/MS) workflows are used to provide comprehensive snapshots of the metabolome (Wolfender *et al.* 2018). However, despite recent advances in computational metabolomics, the reliable annotation of ms/ms spectral data remains a challenge (de Jonge *et al.* 2022). Hence, further complementary analyses, laborious manual annotations, and detailed validations of putative structure hypotheses remain a necessity (Wolfender *et al.* 2018, Beniddir *et al.* 2021). To assist with exploratory data analyses, tools like molecular networking provide with mean to organize and prioritize features for in-depth evaluations (Watrous *et al.* 2012, Nothias *et al.* 2020). From a technical perspective, molecular networking comprises a combination of data clustering and exploratory data visualization. Large datasets are subdivided into smaller, more manageable feature groups via a network topology approach based on spectral similarities (Nothias *et al.* 2020, Mildau *et al.* 2024). Those feature groups are further presented to the user as subnetworks (known as mass spectral molecular families) for exploration of relationships across features within them (Nothias *et al.* 2020). While advantageous as a first analysis step, relationships between subnetworks are lost completely (Olivon *et al.* 2018, Mildau *et al.* 2024), parameter setting for clustering and visualization are time-consuming and their evaluation opaque, and further manual customization of resulting molecular networks in Cytoscape is usually necessary to highlight statistical features for subnetwork or feature prioritization (Pakkir Shah *et al.* 2024).

While statistical data can often be manually integrated into Cytoscape networks, there is currently a lack of workflows in untargeted metabolomics that provide both spectral clustering and statistical data integration on the spectral group level aimed at feature prioritization (McLuskey *et al.* 2021). As a notable exception, the PALS (Pathway Activity Level Scoring) workflow makes use of molecular families, i.e. feature groupings representing “pathways” via linking together chemical analogues, for latent variable summation-based statistical comparisons across treatment groups (Tomfohr *et al.* 2005, McLuskey *et al.* 2021). PALS has two primary limitations however. First, it is based on mass spectral molecular networking groups and thus inherits the time-consuming and opaque clustering approach based on network topology. Second, it is exclusively based on the first component of a latent variable projection of group data, potentially introducing a loss of information. In addition, PALS stops short of visually integrating such analysis results back into the network representations it is based on. The field is thus still lacking an integrated approach providing spectral data clustering, statistical analysis at the group level, and integrated visualization of the results of both of these features for streamlined exploratory data analysis.

**Figure 1.**
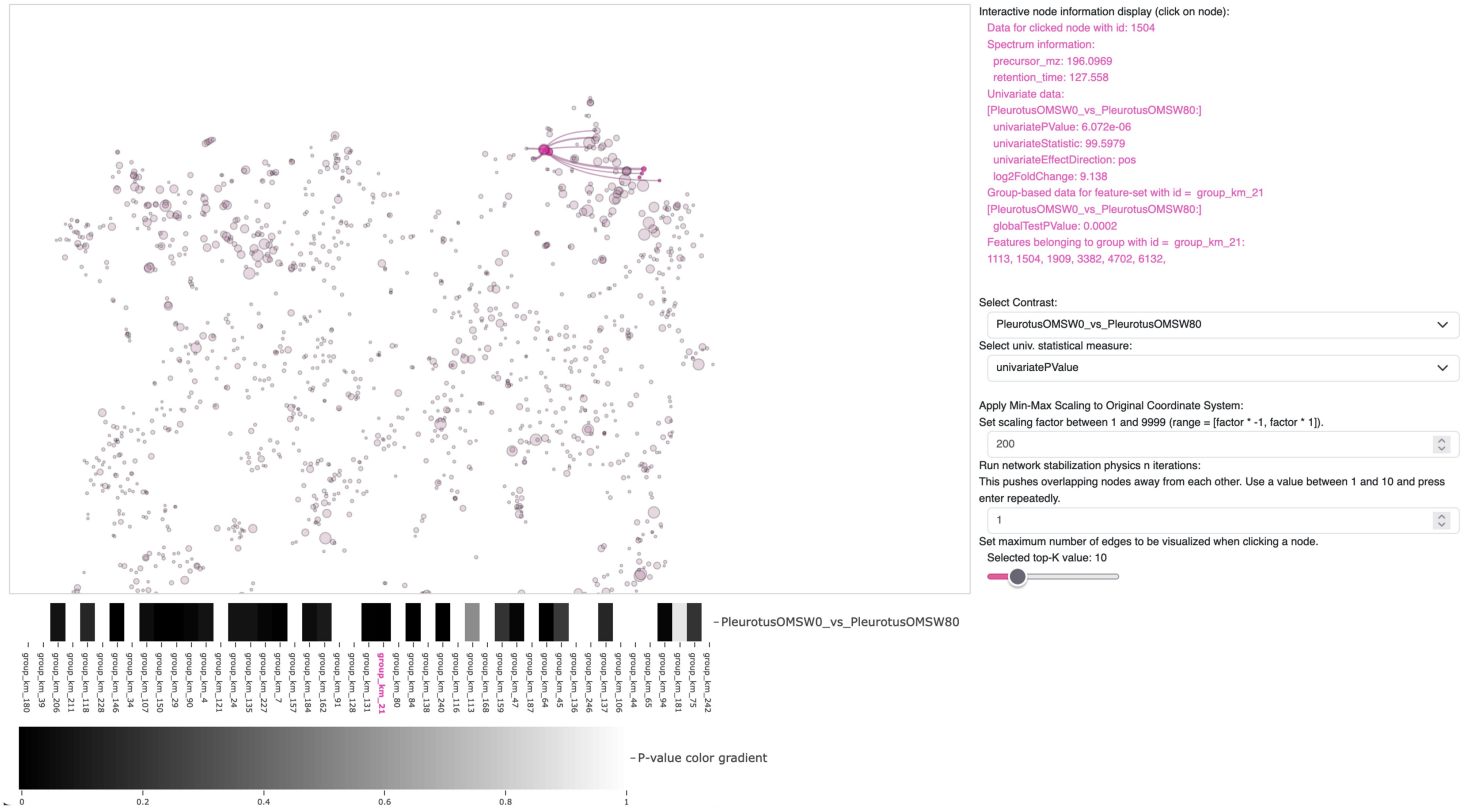
View of the visualization panel of the msFeaST dashboard with overview embedding, side panels, group-wise specific *P*-value heatmap below. A differential feature is selected, highlighting its top-10 neighbors as set, as well as highlighting its set members in color. Node size represents a linear mapping of univariate, feature-specific *P*-values, showing clear areas of enrichment in the t-SNE map.

To fill this gap, we developed **M**ass **S**pectral **Fea**ture **S**et **T**esting (msFeaST), a comprehensive workflow for integrated analysis and visual exploration. We draw inspiration from gene-set testing and pathway enrichment analyses, which shift the focus from the often large and cluttered feature space to aggregated groups such as gene ontologies or pathways. This approach provides the basis group-based comparisons of experimental conditions that can then be further explored in detail (Rosato *et al.* 2018, Chong *et al.* 2020, Maleki *et al.* 2020). To achieve a similar approach in untargeted metabolomics, where genes are replaced by metabolite features with unknown pathway membership, we make use of k-medoids clustering on pairwise similarity data to provide homologous subdivisions of the data (Schubert and Rousseeuw 2021, Mildau *et al.* 2024). Testing is then performed for each cluster using globaltest, providing high power to detect small concordant treatment-specific effects across cluster members via a single group-wise statistical test (Goeman *et al.* 2004). In addition, msFeaST draws visualization inspiration from both specXplore and MetGem making use of a t-SNE embedding to represent the entire spectral dataset in one overview (Maaten 2008, Olivon *et al.* 2018, Mildau *et al.* 2024). This embedding overview, the clustering, and associated cluster-based testing are integrated into an interactive dashboard inspired by EdgeMaps and specXplore, allowing the user to interactively explore and trace pathway relationships between feature nodes within and across clusters in a threshold independent manner (Dork *et al.* 2011, Mildau *et al.* 2024) (Fig. 1).

## 2 Methods and implementation

**Figure 2.**
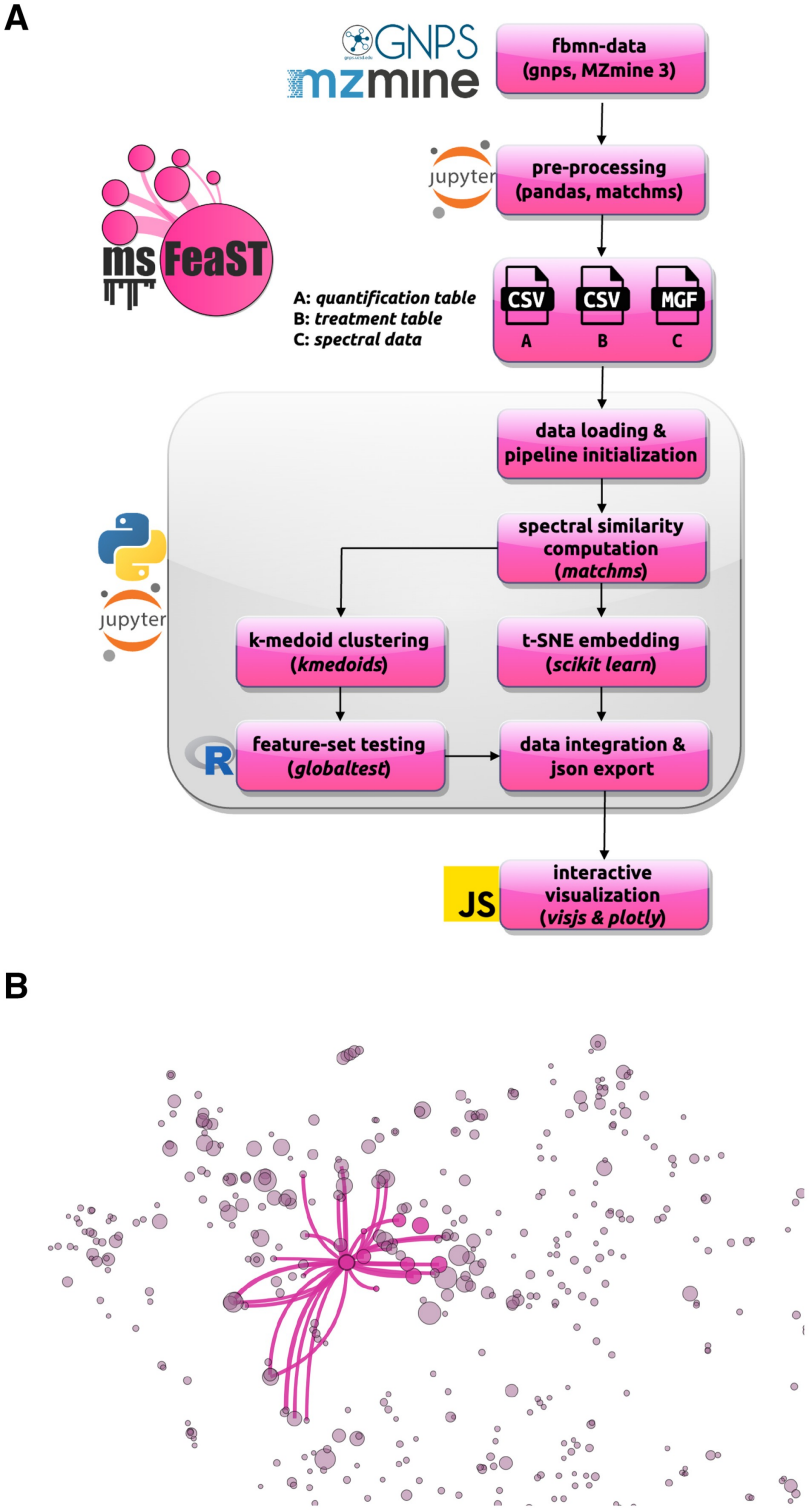
msFeaST workflow overview and dashboard network visualization example. (A) Feature-based molecular networking data is translated into the expected msFeaST input data format. Once loaded, a msFeaST pipeline instance is created. Using msFeaST pipeline methods, spectral similarities are computed, clustering is performed (python package kmedoids), t-SNE embedding is performed (using scikit-learn), feature-set testing is performed (via an embedded R script, globaltest), and data is integrated into a json format compatible with the interactive javascript based visualization (visjs, plotly). The user only needs to run a sequence of commands while intermediate data structures are handled by the pipeline object. (B) msFeaST network visualization example run on the illustrative example mushroom data using ms2deepscore as the scoring approach (Huber *et al*. 2021b; de Jonge *et al*. 2024). Nodes represent individual features, while node size is derived from a linear mapping of statistical properties to node size (e.g. univariate *P*-values, or log-2 fold changes) to visually highlight differential features. The example shows a clicked feature within a feature set (member nodes highlighted in color) that shows differential abundance across fruiting bodies of *Pleurotus eryngii* cultivated using 0% and 80% olive mill solid waste mixed into their substrate. The selected node is shown with its top 30 neighbors, connecting to other clusters (nodes not highlighted in color) within the local t-SNE area.

The msFeaST workflow is a combination of data clustering, statistical testing at the cluster level, and interactive visualization using overlays of 2D embeddings with an ego-network, i.e. a node-centric neighborhood graph (Fig. 1). An overview of the Python and R-based processing pipeline can be seen in Fig. 2.

### 2.1 Data processing for msFeaST

The msFeaST pipeline requires MS/MS spectral data, a feature quantification table, and statistical metadata for two or more sample groups. When working with feature based molecular networking data, pre-processing functionalities are available to load the data accordingly (Schmid *et al.* 2023, Nothias *et al.* 2020). Once the data are loaded, the msFeaST pipeline method is initialized and handles all intermediate data structures. The workflow guides the user through the data processing steps in the form of spectral similarity computations using the desired similarity score (Huber *et al.* 2020, 2021a,b, de Jonge *et al.* 2024), k-medoid clustering based on the resulting distance matrix (Schubert and Rousseeuw 2021, Schubert and Lenssen 2022), and t-SNE embedding using the same structure (Maaten 2008, Gove *et al.* 2022, Lause *et al.* 2024). To set the number of clusters k in clustering, the user may base their decision Silhouette scores for a comprehensive grid of K values, where high values of K are an indicator of better data subdivision (Tibshirani *et al.* 2001). We do note that it is ultimately up to the user to decide on the optimal subsetting of their dataset that best connects with their research question, as any measure such as the Silhouette scores can only be used as a guide (Jain 2010). Similarly, the choice of t-SNE embedding is assisted by a tuning grid over perplexity values with associated distance preservation scores and embedding plotting capabilities for evaluation. We refer to Supplementary Sections S1.1–S1.4 for further details. Here, we will focus specifically on the statistical testing and interactive visualization components.

### 2.2 Statistical testing via globaltest

Within this step of the pipeline the clustered data are passed to a R script for group-wise testing using globaltest, a so-called self-contained null hypothesis test (Goeman *et al.* 2004, Goeman and Bühlmann 2007, Fridley *et al.* 2010, Maleki *et al.* 2020, Goeman and Oosting 2023). The test was originally developed for genetic analyses, but has since found use in the field of targeted metabolomics as well (Rosato *et al.* 2018, Chong *et al.* 2020, Pang *et al.* 2021). The test is based on a generalized linear model which aims to assess whether any of the feature-specific effects in a pre-specified group of features have predictive utility in differentiating treatment groups, a task that is closely related to testing whether treatment group-specific effects are not null (for further details see Supporting Information Section S4). This test does not require concordance of effects but is considered powerful at detecting even small concordant effects (Goeman *et al.* 2004). Within msFeaST, each feature-set and contrast combination, that is control versus (treatment group 1, treatment group 2, etc.), is tested using this model, and *P*-values for the group as a whole, as well as feature-specific univariate *P*-values, are extracted. Multiple testing correction is applied to group-wise *P*-values using the Bonferroni method at the family size given by the number of groups times the number of contrasts. This correction is usually substantially smaller than the correction needed if univariate testing was performed, resulting in less stringent cutoff adjustments. However, special attention is needed when using globaltest with small sample sizes as results may be unreliable (Maleki *et al.* 2019).

### 2.3 Visual integration via interactive dashboard

The results of the msFeaST workflow are integrated into a .json formatted file that can be read by the msFeaST visualization dashboard. The visualization dashboard is run as a local browser-based javascript tool and makes use of the plotly and visjs libraries (Plotly-Technologies-Inc. 2015, VISJS Community 2024) (https://plot.ly/ and https://visjs.org/). Upon opening the website in a modern browser (e.g. Firefox, Chrome, Safari, Edge, etc.), the user may load the generated json file and start their analysis.

Loading the data initiates the interactive visualization tab of the dashboard (Fig. 2). This page contains t-SNE embedding-based overview representation of the spectra data, which serves as both a global data view and interactive exploratory analysis platform. Nodes represent individual features, with their sizes encoding linear mappings to pixel width of feature-specific selectable statistical mappings. Two mappings are available: absolute log-2 fold change for the feature, or feature-specific *P*-values. Both mappings are contrast specific and the user can toggle between contrasts using dropdown menus.

Users may hover over nodes to receive node information, e.g. feature identifiers and group identifier for the group the feature belongs to. In addition, they may click on nodes to receive feature details including feature-specific and group-specific statistics results. The last-clicked node and all members in its k-medoid cluster will be highlighted in color. Clicking on a node further prompts the overlay of local topology via top-K edge drawing, giving a glimpse into connectivity within and across groups of the feature in a similarity threshold independent manner. This approach allows exploring neighborhood for any node regardless of nearest neighbor similarity value. To allow for quick assessments of similarity, edge overlays make use of a discrete similarity mapping to give a qualitative indication of connection strength, while quantitative similarity score labels provide more precise information.

Clicking successively on different nodes adds additional edge overlays until a click on the empty canvas is used to reset edge overlays. In this way, local node neighborhood topology can be explored easily without causing computational bottlenecks or visual overload. In addition to the t-SNE embedding, the tool allows the user to make use of iterations of force-directed layout stabilization to mitigate overlapping nodes sometimes caused by the t-SNE embedding.

Group-based prioritization in the network view is supported by a connected heatmap representation of group-wise *P*-values which provides a quick reference to groups with statistically significant deviations for the selected contrast. Clicking on a group entry in the heatmap highlights the respective group in the t-SNE embedding, and vice-versa.

## 3 Illustrative example

To showcase msFeaST we make use of the high-resolution LC-MS/MS data from the study of Kathib *et al.* (2024). Specifically, we make use of the contrast of the *Pleurotus eryngii* fruiting body samples grown on 0% olive mill solid waste substrate against those grown on 80% olive mill solid waste substrate. While each treatment group contained only three samples, substantial differences in the metabolome can be observed with msFeaST, where many metabolites show elevated log-2 fold changes, and a lower number showing corresponding statistical enrichment patterns. Using the node size encoding, the t-SNE overview representation thus allows to spot in a straightforward manner the features showing differential abundance trends. Feature subsets of potential interest are easily spotted and their local topology can be explored (see Supplementary Fig. S1). For example, the differential feature in Fig. 2B is visualized with its top 30 neighbor nodes, highlighting relatively strong connectivity of this feature beyond its small feature cluster highlighted in color. The displayed edges encode not only connectivity, but also pairwise similarity, providing a means of checking connectivity strength between connected features. The heatmap of globaltest feature-set testing *P*-values provides an overview of cluster-wise results, and a means to highlight promising clusters in the embedding overview 1.

The processed data files are available on github with the respective processing notebooks and can be used to inspect the results within the visualization dashboard. No installation is required for this, only the pre-processed .json file and the html dashboard bundle that can be used using web-browsers are needed (see github readme quickstart). In addition to files for the illustrative example shown here, additional runs using the modified cosine score on the above contrast, as well as a modified cosine score comparison between the different mushroom types are included. The latter show large differential intensity patterns resulting in wide scale feature highlighting.

## 4 Limitations and future work

Our msFeaST workflow combines feature-clustering, feature-set-based statistical analysis, and interactive visualization into one workflow. Each one of these steps comes with potentially impactful choices on the conclusions drawn. We note that k-medoid clustering, its comparison to alternatives, and the formal setting of the parameter k have not been studied in detail. To move beyond the guidelines set and shared in this paper, more research is needed to compare clustering methods and evaluate potential optimality criteria in the context of untargeted metabolomics. We note, however, that strict and clear-cut guidelines are unlikely to be realized. Unsupervised learning problems are generally ill-specified and do not provide singular answers (Jain 2010), nor are untargeted metabolomics mass spectral data or their underlying chemical structures easy to categorize in a universal sense (e.g. Djoumbou Feunang *et al.* 2016, Kim *et al.* 2021).

We make use of globaltest to perform feature-set-based testing. This method is used in pathway enrichment analyses, with the main difference between our tool and existing methods lying in the usage of data-driven feature clustering rather than grouping according to a-priori pathways (Chong *et al.* 2020). A comprehensive study of clustering methods and their interaction with testing (including multiple testing adjustments for feature-sets) and general prioritization metrics would be valuable future research contributions.

We note here that transparency in the use of statistical methods is important for scientific reporting. While we strongly recommend against re-running testing for various k with the aim of increasing the number of significant features, any researcher doing so for exploratory purposes should report the impact of the settings on their final results and conclusions (Steegen *et al.* 2016, Gelman and Hennig 2017). We believe that flexibility of exploratory analyses is critical to the scientific process, and best accommodated with transparency (Tukey 1980, Steegen *et al.* 2016, Gelman and Hennig 2017, Thompson *et al.* 2020). Stricter approaches can and should be used in confirmatory analyses (Wicherts *et al.* 2016).

Finally, we note that visualization strategies employed in msFeaST work for certain ranges of settings, and cannot accommodate all types of data or settings equally well. For example, a large number of clusters, including high significant fraction, may interfere with the heatmap’s capability to pinpoint interesting feature groups. In such a case, most features would be interesting for a follow-up through their strong differential patterns. Extending the visualization capabilities beyond the prioritization and network exploration dashboard is an important area for future developments.

## 5 Conclusion

The msFeaST workflow separates visual design considerations from data subdivision and clustering criteria. Clustering is dealt with separately, using its own optimality criteria, and different clustering methods could in principle be used with the pipeline. It thus allows for a more principled approach to subdividing spectral data that is not impacted by network visualization settings aimed at limiting visual overload (such as edge thresholds, top-K edge limits on each node, maximum cluster sizes). This separation is made possible by making use of a network visualization approach using “interactive details on demand” design. Here, interactive neighborhood explorations using top-K edge overlays provide a robust means of exploring and visualizing data. The visualization and clustering workflows can handle different similarity scores without requiring extensive visual or topological fine tuning. The msFeaST workflow thus seamlessly combines spectral similarity-based feature clustering, feature cluster prioritization using statistical contrast information, and local topological neighborhood exploration in interactive exploratory analysis context. We believe that msFeaST can assist researchers in better understanding their untargeted metabolomics data and identifying relevant chemistry by leveraging both spectral similarity and statistical information.

## Supplementary Material

btae584_Supplementary_Data
