# Supplementary material for: Combined LC-MS/MS feature grouping, statistical prioritization, and interactive networking in msFeaST
Source: Bioinformatics. 2024 Sep 30;40(10):btae584. doi: 10.1093/bioinformatics/btae584 (PMC11471276; doi:10.1093/bioinformatics/btae584)
Supplement: btae584_Supplementary_Data [file btae584_supplementary_data.pdf]

Supplement: Combined LC-MS/MS feature grouping, statistical prioritization, and interactive networking in msFeaST

Kevin Mildau[1,2,3,*], Christoph Büschl[4], Jürgen Zanghellini[2], andJustin J.J. van der Hooft[1,5,*].

September 25, 2024

1 Bioinformatics Group, Wageninen University & Research, Droevendaalsesteeg, 6708PB, Gelderland, The Netherlands
2 Department of Analytical Chemistry, University of Vienna, Währinger Straße,1090, Vienna, Austria
3 Doctoral School in Chemistry (DOSCHEM), University of Vienna, Währinger Straße,1090, Vienna, Austria
4 Institute of Bioanalytics and Agro-Metabolomics, University of Natural Resources and Life Sciences, Konrad-Lorenz-Straße, 3430, Lower Austria, Austria
5 Department of Biochemistry, Auckland Park Kingsway Campus, 2006, University of Johannesburg, Gauteng Province, South Africa
∗ Corresponding authors: kevin.mildau@wur.nl, justin.vanderhooft@wur.nl

1 Further details on the processing steps of the msFeaST workflow

This section contains details on the pre-processing and set-up steps within the msFeaST workflow.

1.1 Data Import

To make use of msFeaST, the user will need three data elements. First, they will need ms/ms spectral data (.mgf format), where each spectral feature has a unique feature identifier. Second, they will need a quantification table, i.e., a table with a sample identifying column, and a column for each feature with corresponding intensity data. Third, they need a treatment table with a sample identifying column and treatment column indicating which sample belongs to which treatment group. Tabular data needs to be provided in .csv format. Feature identifiers, sample identifiers, and treatment identifiers should be unique and consistent across these data structures to ensure successful internal mapping of information. To assist the user with obtaining these data files, msFeaST includes a data pre-processing workflow allowing to parse feature-based molecular networking file exports from GNPS or MZmine 3 (Nothias et al., 2020; Schmid et al., 2023).

1.2 Spectral Similarity Computation

In the first step of the processing pipeline, spectral similarities are computed using matchms (Huber et al., 2020). These may be Modified Cosine Scores, or pre-trained machine-learning-model-based scores such as Spec2Vec or MS2DeepScore 2.0 (Huber et al., 2021a,b; de Jonge et al., 2024). To make sure similarity measures are useful, mass spectral data should be filtered to contain only spectra with sufficient fragment information. Stringent filtering of low information features and spurious fragments will improve the data set quality as a whole by decreasing the sparsity of the similarity matrix.

1.3 K-medoid Clustering

In the second step of the pipeline, the pairwise similarity matrix is transformed to a distance matrix and used to perform k-medoid clustering for different values of k (number of clusters), where the user can select an appropriate number of clusters based on either the optimal Silhouette score or their desired cluster granularity (see figures 1 and 3) (Schubert and Rousseeuw, 2021; Schubert and Lenssen, 2022). Silhouette scores provide an internal cluster validation score based on within cluster and across cluster distances. Specifically, if we assume $a(i)$ to be the average distance from some feature i to other features in its cluster, and $b(i)$ the average distance of feature i to points in the nearest by cluster (the cluster with minimum $b(i)$), then the silhouette statistic is defined by $s(i) = \frac{b(i)-a(i)}{max(a(i),b(i))}$. The Silhouette score is the average silhouette statistic over all features (Tibshirani et al., 2001). Large values are an indication of good clustering performance without the need for any existing class labels.

We note that achieving positive and preferably high Silhouette scores (i.e., a score measuring within cluster similarity against across cluster similarity), implies successful subdivision of the spectral data into more homogeneous subsets. While the number of clusters k can be set, the resulting cluster sizes are determined by the algorithm.

While Silhouette scores may in some cases indicate large numbers of clusters to be optimal, we would recommend against using large numbers of clusters. In the context of the msFeaST prioritization workflow, hundreds of small clusters are inferior to dozens moderately sized clusters. Potential silhouette score improvements should be balanced against increasingly small clusters and thus increasingly little group-wise information. We do note that it is ultimately up to the user to decide on the optimal subsetting of their dataset that best connects with their research question, as any measure such as the Silhouette scores can only be used as a guide (Jain, 2010).

1.4 Embedding using t-SNE

In the third step of the pipeline, t-SNE optimization is run over a range of perplexity values to create a tuning grid for selection of a suitable embedding. Perplexity roughly corresponds to the number of high-dimensional neighbors to be considered in the two-dimensional t-SNE projection. Perplexity has a large influence on the resulting embedding and hence should be selected carefully (Maaten, 2008; Gove et al., 2022). msFeaST provides both Spearman and Pearson correlations between pairwise distances in high-dimensional similarity and 2-dimensional embeddings spaces to guide the selection (e.g. see figure 2). In general, the higher the correlation, the better the distance preservation. However, while distance preservation is a desirable feature, care is needed to make sure visually distinct node groupings are produced in the 2D embedding space (see figures 4a, 4c, 4e and 6). Indeed, the best distance preservation does not necessarily imply best visual representation of inherent group structures (Lause et al., 2024). Alternative embedding approaches such as UMAP or multidimensional scaling (MDS) could also be used with msFeaST, yet were found to be more difficult to tune for the msFeaST visual user case (see figures 4b, 4d, 4f, 7, 8, and 5).

With clustering in hand and embeddings generated, the workflow proceeds with testing and visual integration of results using the msFeaST interactive visualization dashboard.

2 Comparison of msFeaST to molecular networking

The msFeaST workflow involves embedding, clustering, statistical testing, and interactive network visualization in a single workflow. Here, we compare the analysis workflow of msFeaST in more depth with the feature based molecular networking workflow to highlight important shortcomings of the latter. On a technical level molecular networking can be perceived as a two-step data processing and data visualization workflow. In the data processing step, spectra data are turned into a pairwise similarity matrix based on the modified cosine score, which is turned into a collection of subnetworks using various topological settings, among which spectral similarity thresholds, minimum fragment overlaps between pairs, maximum node degree (i.e., maximum number of edges per node, a top-K like setting post threshold cutoffs for edges), and maximum sub-network size (an overall limit to subnetwork size). The combination of these settings is used to create data clusters that can be investigated using network visualization. The networks are fixed in that edges and clustering are determined in pre-processing and displayed by default. It is thus important to use conservative settings to avoid visual clutter through excess connectivity. The main use of this group visualization is to allow for within group pairwise connectivity exploration. While this is a sensible first step organizing datasets for exploration, molecular networking does not come without disadvantages.

Specifically, subdividing networks into strict subnetworks may obscure relationships between such subnetworks or across the spectra they contain. While strict edge cutoffs may be needed for organizing spectral data into disjoint groups using the molecular networking approach, they do represent a loss of topological neighborhood information. In addition, feature grouping is done using a plethora of indirect topological settings, and correspondingly difficult to tune. This is especially true when changing between different scoring approaches with different scoring behavior such as MS2DeepScore as can be demonstrated in specXplore (Mildau et al., 2024). Here, score behavior and dataset specific connectivity trends need to be considered for achieving suitable molecular networking results. Since this is not available in practical applications, users must resort to rerunning molecular networking with different topological settings to assess which settings work for the data at hand. Here, comparison of different settings is non-trivial and laborious given re-ordering and resizing of feature groups and their potential high impacts of small threshold changes on node inter-connectivity, as can be interactively and explored and visualized with specXplore (Mildau et al., 2024). Indeed, small setting differences may easily lead to vast numbers of singleton nodes, i.e., nodes with no inter-connectivity that are useless in pairwise comparisons, or equivalently lead to dense hairball networks with dense inter-connectivity preventing meaningful neighborhood exploration.

In addition to these set-up difficulties, current molecular networking workflows largely rely on the manual visual integration of statistical metadata inside Cytoscape. This integration involves numerous manual steps or requires programmatic data wrangling knowledge (e.g., section 3.5 in Pakkir Shah et al. (2023)). As such, molecular networking is thus better viewed as a starting point for exploratory data analysis workflows rather

than an end-point. The msFeaST workflow improves on the clustering, visualization, and statistical integration workflow components by a) formally separating clustering from visualization and topology, b) including flexible and interactive top-K edge overlays to allow dynamic neighborhood exploration, and c) including direct statistical support for one or more group comparisons.

3 Clustering

While the problem of clustering in metabolomics is far from solved, msFeaST improves on the subdivision approach of molecular networking by i) making the grouping independent of topological and network visualization criteria, ii) providing a rapid approach to trial a large number of subdivisions with corresponding silhouette scores to evaluate and choose suitable subdivision levels. By separating the clustering steps from the visualization itself, we omit the need to simplify the topological connectivity data to accommodate visualization agreeableness criteria such as, e.g., the avoidance of dense hairball networks. Instead, clustering is formally separated, may be exchanged for different clustering approaches in principle, and may use formal internal cluster validation metrics. Clustering is also independent of the t-SNE embedding used since it works directly using the pairwise similarity data. In addition, the use the "overview first, zoom and filter, then details-on- demand" mantra enables seamless interactive inclusion of topological neighborhood representations. Here, highest similarity ranking neighbors for any node can be found, high numbers of edges can be displayed for individual nodes to achieve insights into inter-connectivity including lower scoring similarities, and paths between nodes can be traced by interactively adding edge overlays for subsequently clicked nodes.

By making use of a clustering method that works on arbitrary distance metrics, msFeaST also inherits the flexibility of molecular networking to work in principle with any similarity score provided (provided it can be projected to range [0,1]). Similarity score development and comparison is an active field of research, making this flexibility essential for the workflow to be adaptable to new research insights.

From a general workflow perspective, we note that clustering is used within msFeaST with the primary aim of subdividing the data into more manageable chunks. We consider subdivision to be fruitful as soon as within group homogeneity exceed across group homogeneity, that is, we want the heterogeneous dataset to be subdivided into more homogeneous subsets. How many subsets ought to be used depends on the user, the data (e.g., the system measured, the number of features and their diversity), and the similarity score used. Optimality criteria such as highest overall Silhouette scores can be used to provide formal guidance. However, they should not overturn the intended use scenario. Any internal validation score make use of very specific, yet limited metrics. Achieving optimal Silhouette scores for a dataset at many hundreds of clusters is tantamount to creating many very small clusters, which would not be useful in the exploratory workflow considered here. Indeed, being able to subdivide larger clusters into ever more homogeneous sub-clusters can reduce the degree of overview provided by clustering. More research is needed to determine which internal validation metrics, or combinations of them, would be most suitable to provide a more formal guidance in spectral data sub-division, leaving the setting of K to be a metrically guided but ultimately user determined choice.

While clustering and subdivisions are useful, we note that the homogeneity that can be achieved is domain and data dependent. Here, metabolomics data represent a vast set of highly diverse and overlapping categories of small molecules (metabolites and chemical compounds). This has two implications in that 1) we may always retain relatively high levels of heterogeneity, no matter the approach used unless subdivision become increasingly small, and 2) tradeoffs emphasising different elements of the overlap of lack thereof will lead to fundamentally different but equally valid data subdivisions, as evident by numerous chemical ontologies in the case of known structures.

4 Feature-set-testing

The globaltest method is based on a generalized linear model which aims to assess whether any of the feature-specific effects in a pre-specified group of features have predictive utility in differentiating treatment groups, a task that is closely related to testing whether treatment group-specific effects are not null. The globaltest package makes use of a Generalized Linear Model, i.e.,

$E(Y_i|\beta) = h^{-1}(\alpha + \sum_{j=1}^{m} x_{ij}\beta_j)$,

where α is an intercept, a length m vector of regression coefficients β_j for each feature in the cluster, and a link function h (e.g., identity link). Specifically, the method makes use of the null hypothesis that all feature-specific regression coefficients β_j are 0, i.e.

$H_0 : \beta_1 = \beta_2 = ... = \beta_m = 0.$

To test this hypothesis in the situation where the number of features m is larger than the number of samples n, globaltest assumes stochastic regression coefficients drawn from a common distribution with an expectation of zero and variance of τ^2. Assuming this, the null hypothesis can be rephrased in terms of this variance component as

$H_0 : \tau^2 = 0.$

Testing against this null hypothesis is done using a score test and returns a single p-value for a group of features aimed at detecting whether any of the features within the group showed differential intensity. We note that the globaltest method has also found use in the metabolomics community via its inclusion in the Metaboanalyst targeted metabolomics workflow (Pang et al., 2021; Chong et al., 2020).

Statistical testing in msFeaST is based on globaltest and thus critically relies on the provision of feature group membership. Since msFeaST is an Exploratory Data Analysis tool for largely unknown feature spaces, these groupings are based on the data themselves. Importantly, the information used for statistical analyses using hypothesis testing should be orthogonal to the information used for grouping. This needs to be the case to avoid selection steps biasing the calibration of p-values. Indeed, if magnitude of effect were used to form feature groups, a few highly impressive groups can be expected to be created even for data with no inherent grouping or treatments effects at all. The globaltest approach does not inherently protect against such biasing selection effects on its own, nor are the correction factors that could be used to deal with this. By contrast, the choice of basing the subdivision on mass spectral similarity data, which stands independent of precursor intensity patterns across samples (except for minimum intensity requirements of mass signals to be retained), does not introduce biasing selection effects. The use of such orthogonal information layers is functionally equivalent to mapping genes or known metabolites to pathways based on their sequence or structural information.

5 Gatekeeping and descriptive use of p-values in msFeaST

The p-values by globaltest are used in two fundamentally different ways within msFeaST. First, they provide a means of splitting the data into priority and non-priority feature groups via determination the statistical significance of group level trends. Second, p-values are used in a descriptive fashion as a measure of group-level or feature-level discordance from the null hypothesis to rank groups in order of interest. For validity of the gatekeeping role in the first use case it is important to avoid biasing selection effect from invalidating the p-values generated. In general, rerunning analyses with different k to optimize for larger numbers of significant groups or pre-selecting feature groups using treatment specific intensity patterns can severely impact the operating characteristic of testing processes and should be avoided. Users should perform and evaluate the clustering task separately from the statistical testing based on it. The descriptive approach makes no claims with regards to operating characteristics of tests. It is merely used for ranking feature-sets and thus unaffected by testing considerations as no claims are made with respect to how many of the top-most features are to be considered statistically significant or not. Moreover, since globaltest p-values only provide limited insight into feature-specific (or group-specific) effect magnitudes, log 2 fold change descriptive statistics are additionally computed for each individual feature for inclusion in visual analyses.

6 msFeaST relationship to PALS (Pathway Activity Level Scoring)

The authors are aware of one alternative workflow merging spectral grouping and group level statistical information in untargeted metabolomics in the form of PALS (Pathway Activity Level Scoring) (McLuskey et al., 2021; Tomfohr et al., 2005). The msFeaST workflow has resemblances to PALS which uses the mPLAGE (PLAGE for metabolomics) method to score feature-set differential intensity across treatment groups. Like globaltest in msFeaST, the mPLAGE method also makes use of a so-called self-contained null hypothesis. However, the latter is based on summarizing group data using latent variables rather than random effect model based. The core differences between PALS and msFeaST are the following: i) msFeaST makes use of globaltest as the core statistical package underlying the feature set-testing, avoiding the need for latent variable projections and corresponding tuning choices (Fridley et al., 2010), ii) msFeaST avoids the molecular networking dependency by providing its own clustering and cluster number tuning approach, and iii) msFeaST integrates the statistical results into its own interactive network visualization environment.

7 Interactive Visualization & Automatic-Visual-Constraining

The visualization approach of msFeaST focuses on two elements. First, it provides a two-dimensional overview of the whole dataset using a two-dimensional t-SNE-based scatterplot approach. Nodes represent individual spectral features, and node sizes are a determined via a mapping of feature-specific descriptive statistics. Large node sizes correspond to larger group difference, providing a means of quickly spotting differential nodes. Second, it provides a means of exploring local topology in a threshold independent manner. For any node, i.e., marker in the scatterplot, clicking the node reveals its cluster via a color highlight, and its top-k neighbors via edge overlays. The edge overlays make use of a discrete edge weight encoding and quantitative label to give clear indications of the effective similarity between the connected features. The top-K parameter can be adjusted dynamically in a range spanning from 1 to 50, allowing inspection of close and more distant neighbors in the graph up to the pragmatic limit of 50. Higher top-K limits are technically possible, but seem impractical. Clicking on nodes successively allows adding ego-network representations for more and more nodes, effectively

creating local network representations. Complementing network overlays are hover tooltips and node information textual displays providing more in-depth information about the feature and its group. Using this simple set of interactivity, the user can navigate local topology and inspect feature groups of interest, as well as their relationship with features across the network.

Since the overview and interactivity are feature-centric by design, a group-centric heatmap representation is added to afford an overview of group-level statistical results. Clicking or hovering over the heatmap highlights the corresponding group in the overlay representation, thus providing a means to navigate from group-level to feature level. In addition to node and group interactive components, additional selection and setting panels can be controlled via click selections (i.e., selection of contrasts or statistical measures encoding node size), or entering new settings and pressing enter on the keyboard (i.e., performing force-directed layout adjustment or resizing the network).

Visualization choices in msFeaST aim towards effective prioritization and automatization. For instance, while node size is used to encode statistical measures, it does so using strictly bounded ranges, e.g., the range of node size 10 to 50 is linearly mapped to p-values between 0 and 6 on a log10 scale, any values outside of this range a flattened to the nearest border node size of 10 or 50 respectively. This has to be done to avoid individual outlying values taking over the entire visual dynamic range with the user not having any means of adjusting the views. Such adjustments are commonly done in scientific visualizations. For instance, when displaying volcano plots with individual outliers flatting the whole representation, outliers may be removed to allow inspection of the remainder of the dataset. Since the msFeaST overview network representation is an interactable exploration platform, removal is not an option. Bounding provides an effective means of constraining the visual range that does not interfere with the goals of prioritization. Any distortions resulting from bounding effects in msFeaST are offset by the ability to inspect the exact quantitative values by interactively prompting quantitative details. An additional visual constraint implemented is that feature-groups are not visually highlighted by default. Instead, click interactivity is required to highlight individual feature groups in the data. This is done to avoid visual overload from highlighting (e.g., color-based) of a potentially very large set of feature-groups at once.

8 Limitations of the msFeaST workflow & future work

The msFeaST pipeline makes use of non-traditional network visualization approaches requiring interactive elements to work. Rendering the whole network data with top-K neighbors for all nodes would quickly become visually overwhelming, even for low top-K values. It is hence not possible to export the network visualizations in a traditional molecular networking sense. However, t-SNE embeddings form a data representation in their own right, and may be used for displaying the whole or parts of the data. The development of cluster (visualization) exports would represent promising avenue for future work.

While globaltest is a powerful approach to the statistical detection of differential intensity patterns in grouped data, it lacks an intuitive output scale. The p-values give no direct indication of the magnitude of effect, and set-level analyses may obfuscate the effects leading to significance which may be consistent and concordant effects across many features, or individual features with strong differential patterns within the groups, where both situations may represent relevant biochemical scenarios (Rosato et al., 2018). In addition, aggregate test p-values give no tangible idea of effect direction on the group level. Naturally, which features are included in sets and the size of sets may have an impact on the nature of set level analyses too. This means that, while testing can be a useful criterion for the delineation of aggregate level statistical anomalies, it does ultimately provide limited insights beyond this. This then necessitates feature-level assessments or additional supporting group-level descriptives to more fully interpret results. The msFeaST pipeline provides some insights into this via encoding of feature-specific effects into the network visualization. However, more specialized visualizations for quantitative inspection such as heatmaps may provide clearer visual representations. On a group-level, additional descriptive statistics summarizing group-level effects from different angles such as concordance of effects in direction and magnitude, overall average magnitude of effect, and individual outlier effect impact, may be useful workflow additions to provide further nuance to group-based analysis.

The msFeaST workflow provides a streamlined and automatic workflow incorporating statistical testing. When incorporated into automatic analysis workflows, the latter becomes convenient and easy to use, yet also untransparent. We reason that it is more difficult for end users to fully appreciate the modeling used, and impossible to inspect the model assumptions and its potential violations and their impact from within a pipeline such as msFeaST. Ultimately, scientists will have to delve deeper into their preferred methodology to do additional quality control evaluations. Within the Exploratory Data Analysis confines of msFeaST, these repercussions may be relatively harmless, yet from a formal testing point of view incorporation of more elaborate misspecification diagnostics would be crucial to guarantee proper operating characteristics of testing procedures.

The current msFeaST workflow provides a streamlined means of performing one particular Exploratory Data Analysis routine. The interactive visualization is more limited than the one we have previously developed in specXplore, and the translation of specXplore visual capabilities to msFeaST, including spectral plots and fragmentation overview maps, may significantly improve the tool's comprehensiveness with respect to mass spectral data exploration capacities. From a visualization perspective, the use of node size to encode effect

provides a conundrum in that positive and negative effects are displayed identically, potentially obfuscating positive or negative concordance or discordance. A means of highlighting positive or negative effect within the network view may useful. In addition, metadata inclusion capabilities for display within the tool itself could be useful to limit the user's need to work within multiple tools at the same time. For instance, putative structure predictions and their visualization within the dashboard would allow streamlined information inspection that currently requires switching between tools using feature identifiers. Moreover, support for additional similarity scores, for additional embedding approaches, and additional clustering methods may make the workflow more suitable for the needs of different researchers. Finally, inclusion of additional feature-set level and feature-specific statistical measures would allow the workflow to cover a broader range experimental designs and effect prioritization considerations.

9 Other work bypassing metabolite identification through pathway enrichment analysis

A core assumption in msFeaST, and in fact any set-testing approach, is that the feature groupings provide scientifically meaningful collections. We argue that this is a reasonable assumption in metabolomics, where many metabolites are inherently linked to each other via shared synthesis pathways or reaction networks. Examples of tools that makes use of this linking are the previously described molecular networking (Watrous et al., 2012; Nothias et al., 2020), but also mummichog (Lu et al., 2022). In mummichog, a library mismatch robust approach to pathway enrichment was developed via local network enrichment profiling. The mummichog pipeline makes use of the best ranking library matches for each compound (true and false) and maps their enrichment to metabolic pathway networks. False matches are assumed to map to completely random locations in the network, thus bearing no systematic relation to metabolic pathways. This means that mismatch based activation will be represented by randomly scattered noise. True matches however, will tend to cluster to the actual enriched pathways, providing a means of identifying elevated pathway activity despite mismatches through local network enrichment patterns (Lu et al., 2022).

10 Supplementary Figures

This section collects supplementary figures in static form. Interactive versions of these figures can be found on github under `https://github.com/kevinmildau/msFeaST/tree/feedback_inclusion/notebooks/data/omsw_pleurotus_ms2deepscore`. The interactive figures allow inspection of the plots in more detail, and provide access to node information via hover information boxes.

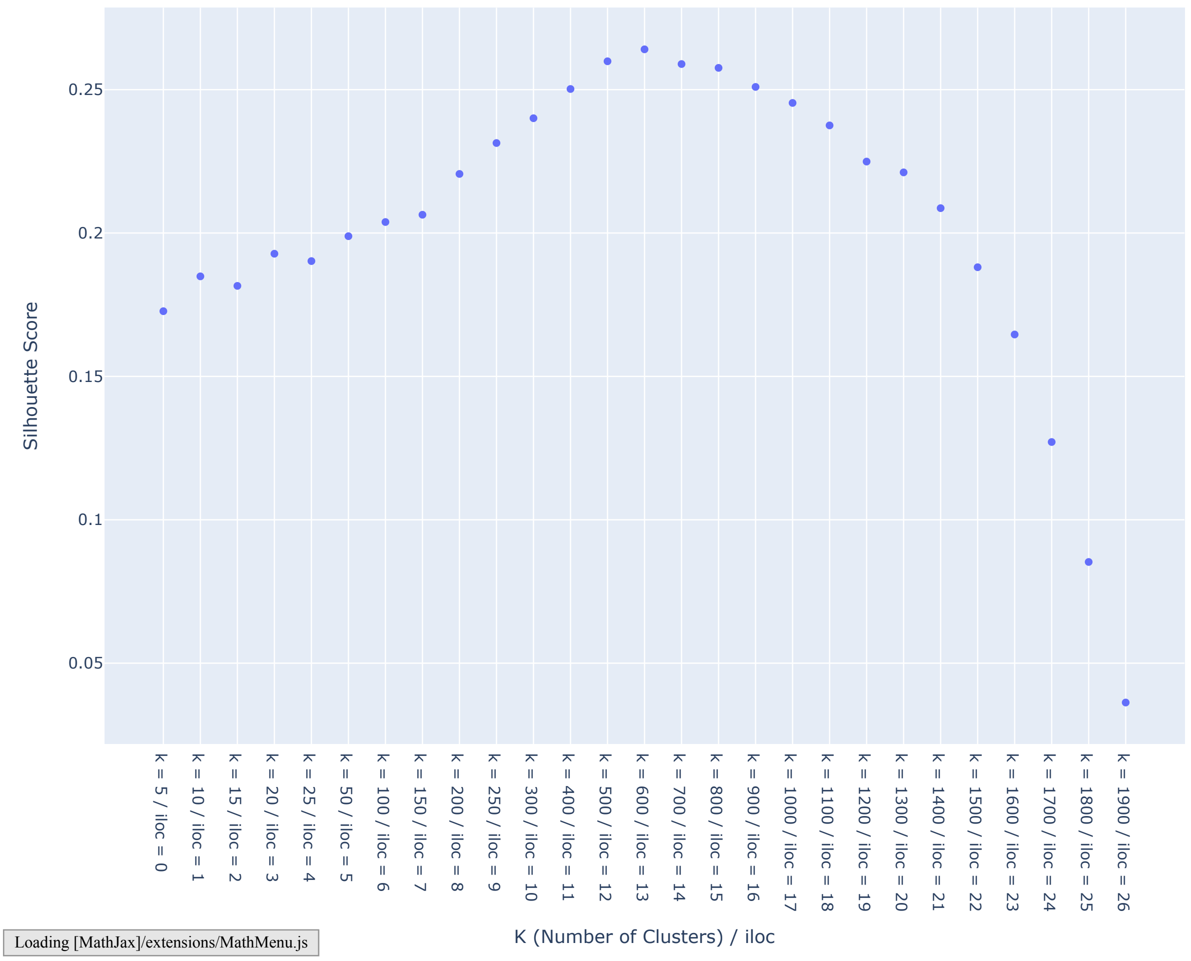

Loading [MathJax]/extensions/MathMenu.js

Figure 1: Silhouette scores for a wide range of k in k-medoid clustering of the Pleurotus ms2deepscore example data. For the range of k considered, $k = 600$ leads to the best Silhouette score. We note however that subdividing the data into 600 clusters leads to many very small clusters. While less optimal from the perspective of the Silhouette score, choosing a value of k below 300 is advisable for purposes of data organization.

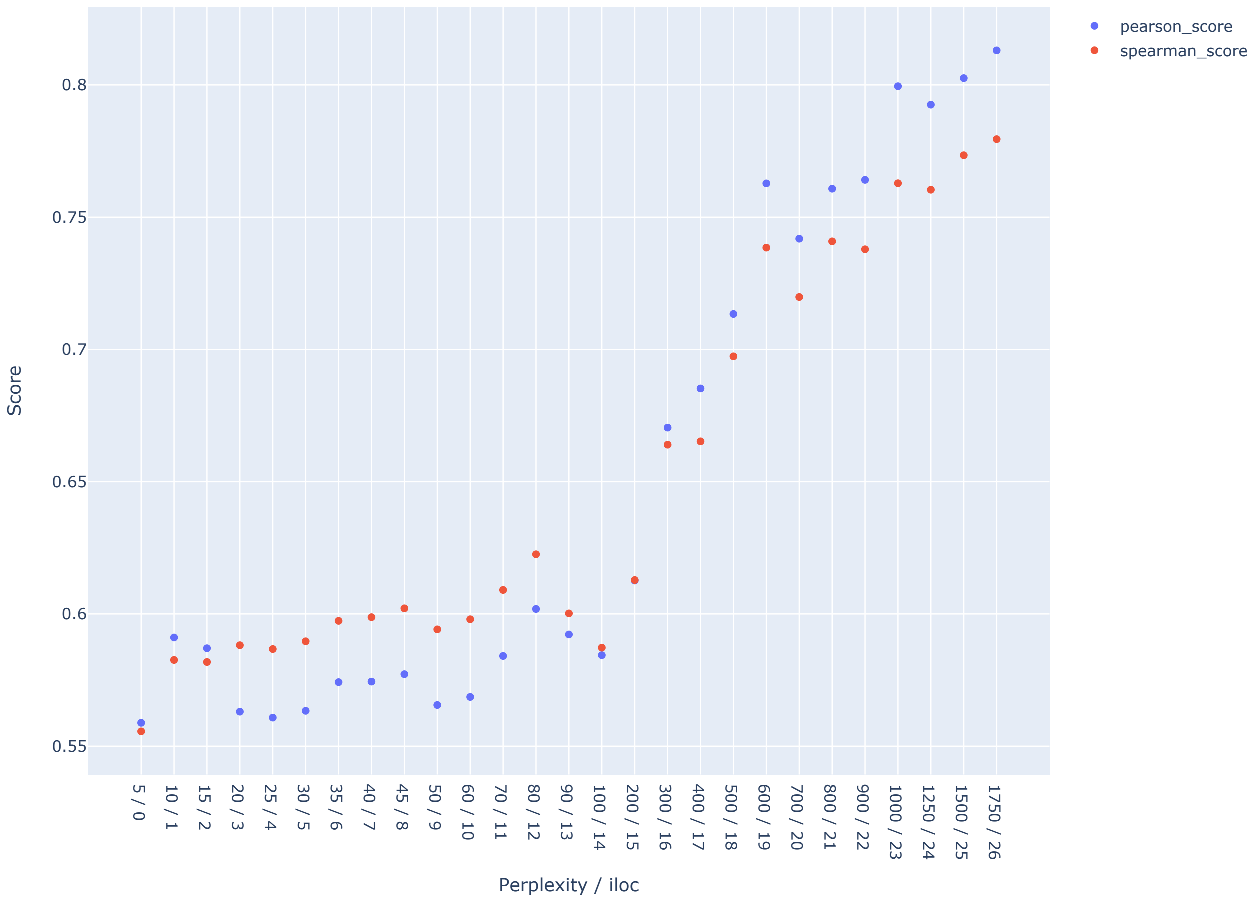

Figure 2: Pearson and Spearman correlations between the MS2Deepscore pairwise distances between features and Euclidean pairwise distances in the t-SNE two-dimensional embedding. Correlations shown for a wide range of perplexity values. We note that distance preservation remains fairly stable until perplexity values above 200, at which point distance preservation improves markedly. Here, much larger features neighborhoods are considered in the embedding algorithm, leading to an increased focus on global distances.

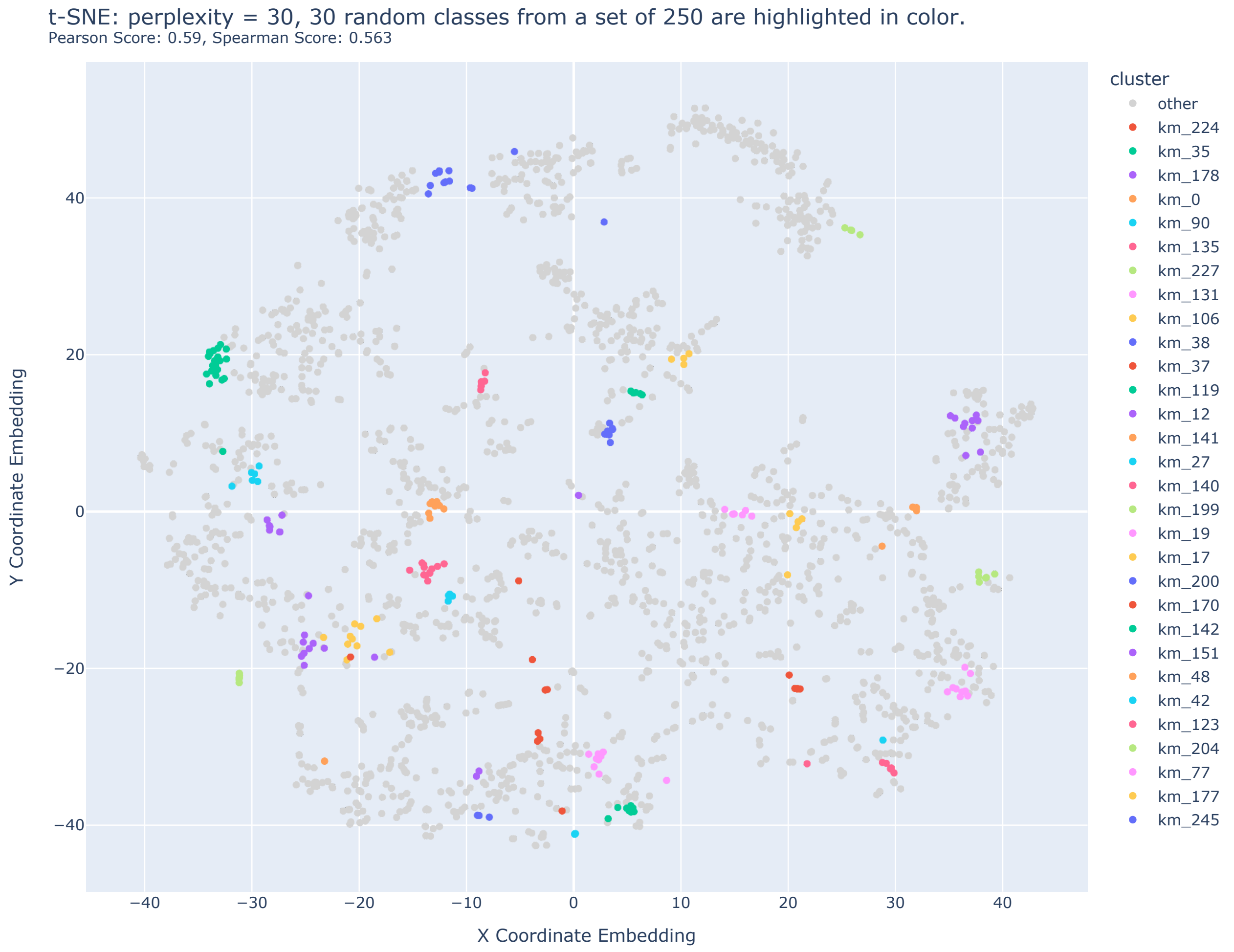

Figure 3: A t-SNE embedding of the ms2deepscore Pleurotus example data. Perplexity was set to 30. Thirty randomly selected clusters from a k-medoids clustering with k = 250 are superimposed in color, where the number of clusters is limited to 30 to avoid visual overload. The superimposed clusters tend to occupy local neighborhoods in the t-SNE embedding.

References

J. Chong, P. Liu, G. Zhou, and J. Xia. Using microbiomeanalyst for comprehensive statistical, functional, and meta-analysis of microbiome data. *Nature Protocols*, 15(3):799–821, Jan. 2020. ISSN 1750-2799. doi: 10.1038/s41596-019-0264-1. URL `http://dx.doi.org/10.1038/s41596-019-0264-1`.

N. de Jonge, D. Joas, L.-J. Truong, J. J. van der Hooft, and F. Huber. Reliable cross-ion mode chemical similarity prediction between ms2spectra, Mar. 2024. URL `http://dx.doi.org/10.1101/2024.03.25.586580`.

B. L. Fridley, G. D. Jenkins, and J. M. Biernacka. Self-contained gene-set analysis of expression data: An evaluation of existing and novel methods. *PLoS ONE*, 5(9):e12693, Sept. 2010. ISSN 1932-6203. doi: 10.1371/journal.pone.0012693. URL `http://dx.doi.org/10.1371/journal.pone.0012693`.

R. Gove, L. Cadalzo, N. Leiby, J. M. Singer, and A. Zaitzeff. New guidance for using t-sne: Alternative defaults, hyperparameter selection automation, and comparative evaluation. *Visual Informatics*, 6(2):87–97, June 2022. ISSN 2468-502X. doi: 10.1016/j.visinf.2022.04.003. URL `http://dx.doi.org/10.1016/j.visinf.2022.04.003`.

F. Huber, S. Verhoeven, C. Meijer, H. Spreeuw, E. Castilla, C. Geng, J. van der Hooft, S. Rogers, A. Belloum, F. Diblen, and J. Spaaks. matchms - processing and similarity evaluation of mass spectrometry data. *Journal of Open Source Software*, 5(52):2411, Aug. 2020. ISSN 2475-9066. doi: 10.21105/joss.02411. URL `http://dx.doi.org/10.21105/joss.02411`.

F. Huber, L. Ridder, S. Verhoeven, J. H. Spaaks, F. Diblen, S. Rogers, and J. J. J. van der Hooft. Spec2vec: Improved mass spectral similarity scoring through learning of structural relationships. *PLOS Computational Biology*, 17(2):e1008724, Feb. 2021a. ISSN 1553-7358. doi: 10.1371/journal.pcbi.1008724. URL `http://dx.doi.org/10.1371/journal.pcbi.1008724`.

F. Huber, S. van der Burg, J. J. J. van der Hooft, and L. Ridder. Ms2deepscore: a novel deep learning similarity measure to compare tandem mass spectra. *Journal of Cheminformatics*, 13(1), Oct. 2021b. ISSN 1758-2946. doi: 10.1186/s13321-021-00558-4. URL `http://dx.doi.org/10.1186/s13321-021-00558-4`.

A. K. Jain. Data clustering: 50 years beyond k-means. *Pattern Recognition Letters*, 31(8):651–666, June 2010. ISSN 0167-8655. doi: 10.1016/j.patrec.2009.09.011. URL `http://dx.doi.org/10.1016/j.patrec.2009.09.011`.

J. Lause, D. Kobak, and P. Berens. The art of seeing the elephant in the room: 2d embeddings of single-cell data do make sense, Mar. 2024. URL `http://dx.doi.org/10.1101/2024.03.26.586728`.

Y. Lu, Z. Pang, and J. Xia. Comprehensive investigation of pathway enrichment methods for functional interpretation of lc–ms global metabolomics data. *Briefings in Bioinformatics*, 24(1), Dec. 2022. ISSN 1477-4054. doi: 10.1093/bib/bbac553. URL `http://dx.doi.org/10.1093/bib/bbac553`.

L. Maaten. Visualizing data using t-sne. *Journal of machine learning research*, 9(Nov):2579, 2008.

K. McLuskey, J. Wandy, I. Vincent, J. J. J. van der Hooft, S. Rogers, K. Burgess, and R. Daly. Ranking metabolite sets by their activity levels. *Metabolites*, 11(2):103, Feb. 2021. ISSN 2218-1989. doi: 10.3390/metabo11020103. URL `http://dx.doi.org/10.3390/metabo11020103`.

K. Mildau, H. Ehlers, I. Oesterle, M. Pristner, B. Warth, M. Doppler, C. Bueschl, J. Zanghellini, and J. J. J. van der Hooft. Tailored mass spectral data exploration using the specxplore interactive dashboard. *Analytical Chemistry*, Apr 2024. ISSN 0003-2700. doi: 10.1021/acs.analchem.3c04444. URL `https://doi.org/10.1021/acs.analchem.3c04444`.

L.-F. Nothias, D. Petras, R. Schmid, K. Dührkop, J. Rainer, A. Sarvepalli, I. Protsyuk, M. Ernst, H. Tsugawa, M. Fleischauer, F. Aicheler, A. A. Aksenov, O. Alka, P.-M. Allard, A. Barsch, X. Cachet, A. M. Caraballo-Rodriguez, R. R. Da Silva, T. Dang, N. Garg, J. M. Gauglitz, A. Gurevich, G. Isaac, A. K. Jarmusch, Z. Kameník, K. B. Kang, N. Kessler, I. Koester, A. Korf, A. Le Gouellec, M. Ludwig, C. Martin H., L.-I. McCall, J. McSayles, S. W. Meyer, H. Mohimani, M. Morsy, O. Moyne, S. Neumann, H. Neuweger, N. H. Nguyen, M. Nothias-Esposito, J. Paolini, V. V. Phelan, T. Pluskal, R. A. Quinn, S. Rogers, B. Shrestha, A. Tripathi, J. J. J. van der Hooft, F. Vargas, K. C. Weldon, M. Witting, H. Yang, Z. Zhang, F. Zubeil, O. Kohlbacher, S. Böcker, T. Alexandrov, N. Bandeira, M. Wang, and P. C. Dorrestein. Feature-based molecular networking in the gnps analysis environment. *Nature Methods*, 17(9):905–908, Aug. 2020. ISSN 1548-7105. doi: 10.1038/s41592-020-0933-6. URL `http://dx.doi.org/10.1038/s41592-020-0933-6`.

A. K. Pakkir Shah, A. Walter, F. Ottosson, F. Russo, M. Navarro-Díaz, J. Boldt, J.-C. Kalinski, E. E. Kontou, J. Elofson, A. Polyzois, C. González-Marín, S. Farrell, M. R. Aggerbeck, T. Pruksatrakul, N. Chan, Y. Wang, M. Pöchhacker, C. Brungs, B. Cámara, A. M. Caraballo-Rodríguez, A. Cumsille, F. de Oliveira, K. Dührkop,

Y. El Abiead, C. Geibel, L. G. Graves, M. Hansen, S. Heuckeroth, S. Knoblauch, A. Kostenko, M. C. Kuijpers, K. Mildau, S. Papadopoulos Lambidis, P. W. Portal Gomes, T. Schramm, K. Steuer-Lodd, P. Stincone, S. Tayyab, G. A. Vitale, B. C. Wagner, S. Xing, M. T. Yazzie, S. Zuffa, M. de Kruijff, C. Beemelmanns, H. Link, C. Mayer, J. J. van der Hooft, T. Damiani, T. Pluskal, P. C. Dorrestein, J. Stanstrup, R. Schmid, M. Wang, A. T. Aron, M. Ernst, and D. Petras. The hitchhiker's guide to statistical analysis of feature-based molecular networks from non-targeted metabolomics data, Nov. 2023. URL http://dx.doi.org/10.26434/chemrxiv-2023-wwbt0.

Z. Pang, J. Chong, G. Zhou, D. A. de Lima Morais, L. Chang, M. Barrette, C. Gauthier, P.-E. Jacques, S. Li, and J. Xia. Metaboanalyst 5.0: narrowing the gap between raw spectra and functional insights. *Nucleic Acids Research*, 49(W1):W388–W396, May 2021. ISSN 1362-4962. doi: 10.1093/nar/gkab382. URL http://dx.doi.org/10.1093/nar/gkab382.

A. Rosato, L. Tenori, M. Cascante, P. R. De Atauri Carulla, V. A. P. Martins dos Santos, and E. Saccenti. From correlation to causation: analysis of metabolomics data using systems biology approaches. *Metabolomics*, 14 (4), Feb. 2018. ISSN 1573-3890. doi: 10.1007/s11306-018-1335-y. URL http://dx.doi.org/10.1007/s11306-018-1335-y.

R. Schmid, S. Heuckeroth, A. Korf, A. Smirnov, O. Myers, T. S. Dyrlund, R. Bushuiev, K. J. Murray, N. Hoffmann, M. Lu, A. Sarvepalli, Z. Zhang, M. Fleischauer, K. Dührkop, M. Wesner, S. J. Hoogstra, E. Rudt, O. Mokshyna, C. Brungs, K. Ponomarov, L. Mutabdžija, T. Damiani, C. J. Pudney, M. Earll, P. O. Helmer, T. R. Fallon, T. Schulze, A. Rivas-Ubach, A. Bilbao, H. Richter, L.-F. Nothias, M. Wang, M. Orešič, J.-K. Weng, S. Böcker, A. Jeibmann, H. Hayen, U. Karst, P. C. Dorrestein, D. Petras, X. Du, and T. Pluskal. Integrative analysis of multimodal mass spectrometry data in mzmine 3. *Nature Biotechnology*, 41(4):447–449, Mar. 2023. ISSN 1546-1696. doi: 10.1038/s41587-023-01690-2. URL http://dx.doi.org/10.1038/s41587-023-01690-2.

E. Schubert and L. Lenssen. Fast k-medoids clustering in rust and python. *Journal of Open Source Software*, 7 (75):4183, July 2022. ISSN 2475-9066. doi: 10.21105/joss.04183. URL http://dx.doi.org/10.21105/joss.04183.

E. Schubert and P. J. Rousseeuw. Fast and eager k-medoids clustering: O(k) runtime improvement of the pam, clara, and clarans algorithms. *Information Systems*, 101:101804, Nov. 2021. ISSN 0306-4379. doi: 10.1016/j.is.2021.101804. URL http://dx.doi.org/10.1016/j.is.2021.101804.

R. Tibshirani, G. Walther, and T. Hastie. Estimating the number of clusters in a data set via the gap statistic. *Journal of the Royal Statistical Society Series B: Statistical Methodology*, 63(2):411–423, July 2001. ISSN 1467-9868. doi: 10.1111/1467-9868.00293. URL http://dx.doi.org/10.1111/1467-9868.00293.

J. Tomfohr, J. Lu, and T. B. Kepler. Pathway level analysis of gene expression using singular value decomposition. *BMC Bioinformatics*, 6(1), Sept. 2005. ISSN 1471-2105. doi: 10.1186/1471-2105-6-225. URL http://dx.doi.org/10.1186/1471-2105-6-225.

J. Watrous, P. Roach, T. Alexandrov, B. S. Heath, J. Y. Yang, R. D. Kersten, M. van der Voort, K. Pogliano, H. Gross, J. M. Raaijmakers, B. S. Moore, J. Laskin, N. Bandeira, and P. C. Dorrestein. Mass spectral molecular networking of living microbial colonies. *Proceedings of the National Academy of Sciences*, 109 (26), May 2012. ISSN 1091-6490. doi: 10.1073/pnas.1203689109. URL http://dx.doi.org/10.1073/pnas.1203689109.

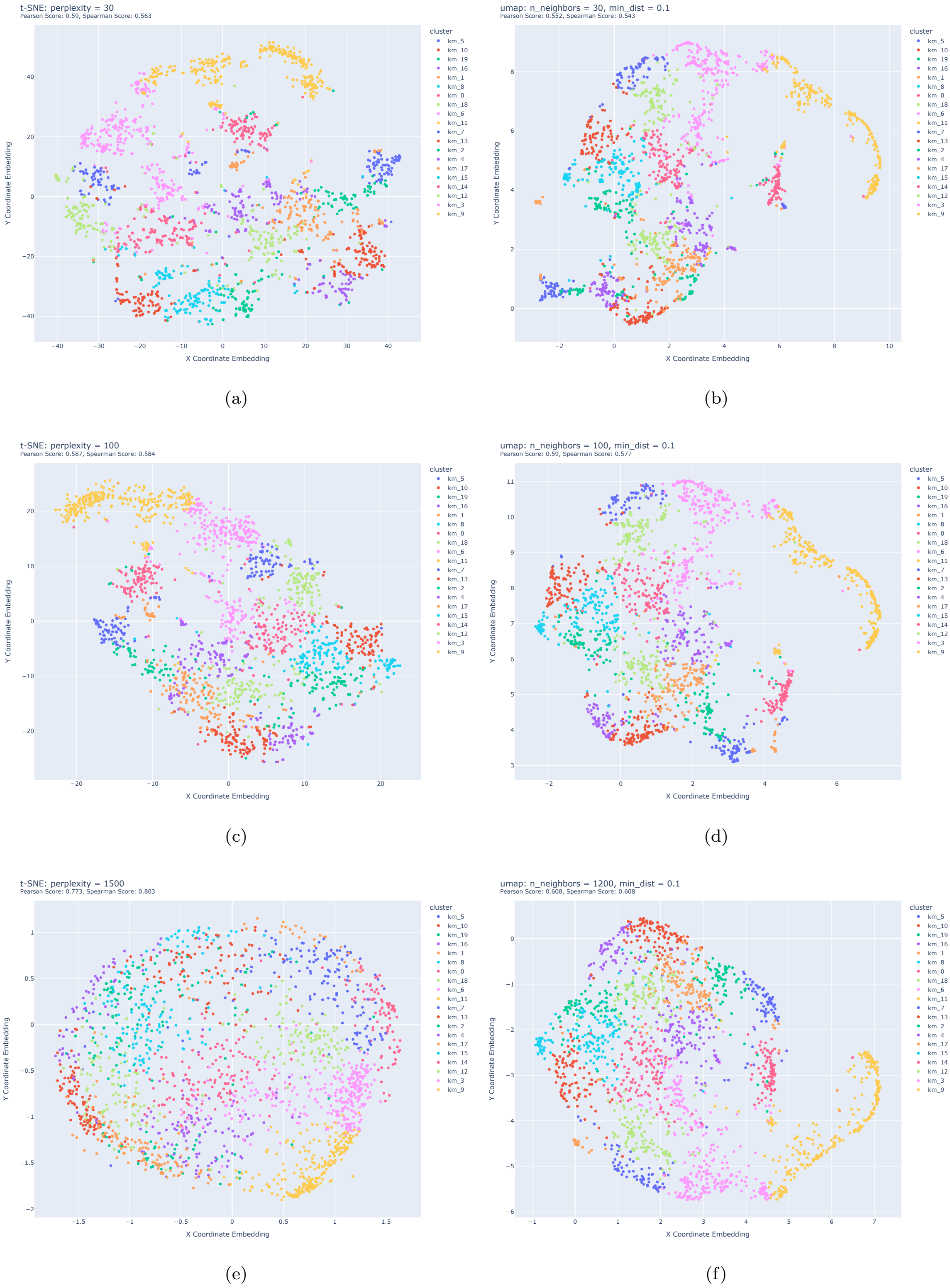

Figure 4: A collection of t-SNE and umap embeddings of the ms2deepscore Pleurotus example data for different algorithm parameters. The number of neighbors considered in embedding is increased from 30 to over 1000 for both t-SNE and umap. As neighborhood size increases, the embedding becomes better from distance preservation perspective, yet fails to produce clusters with clear spatial separation. Overlaps between the different clusters increase, where the whole datasets appears to be placed into one large point cloud requiring color overlays to see any apparent patterns. The umap embedding method further has a tendency to produce dense point ridges that can make network edge overlays difficult to read. The figures display a) a t-SNE embedding with perplexity set to 30, where clear cluster separation can be observed, b) a umap embedding with n-neighbors set to 30, where clear cluster separation can be observed, alongside dense point ridges, c) a t-SNE embedding with perplexity set to 100, where cluster separation without color overlays would be much more difficult, d) a umap embedding with n-neighbors set to 100, where cluster separation is poorer than for lower values of n-neighbors and point ridges remain, e) a t-SNE embedding with perplexity set to 1500, where clusters lack clear separation from one another without the color overlay, and f) a umap embedding with n-neighbors set to 1200, where clusters lack clear separation from one another without the color overlay, with some point ridges remaining.

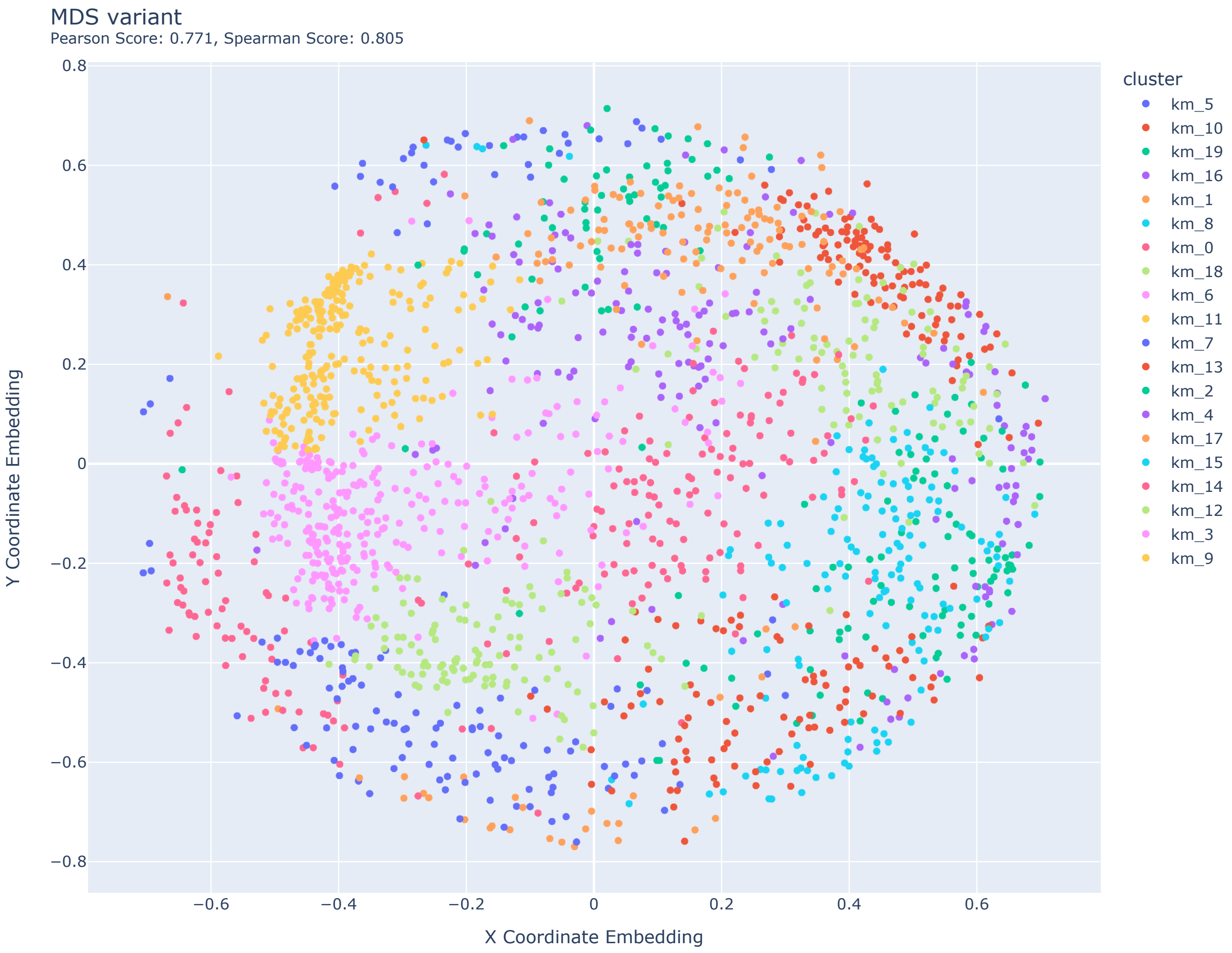

Figure 5: A multidimensional scaling embedding of the ms2deepscore Pleurotus example data. The data was subdivided into 20 clusters using k-medoids clustering on the ms2deepscore derived pairwise distance matrix and not on the two-dimensional embedding. Clusters are highlighted in color. Clusters can be distinguished via their color but heavily bleed into one another. Without color overlays any apparent structure would be difficult to extract. Distance preservation scores are high. Results are qualitative similar to t-SNE with high perplexity (figure 4e)

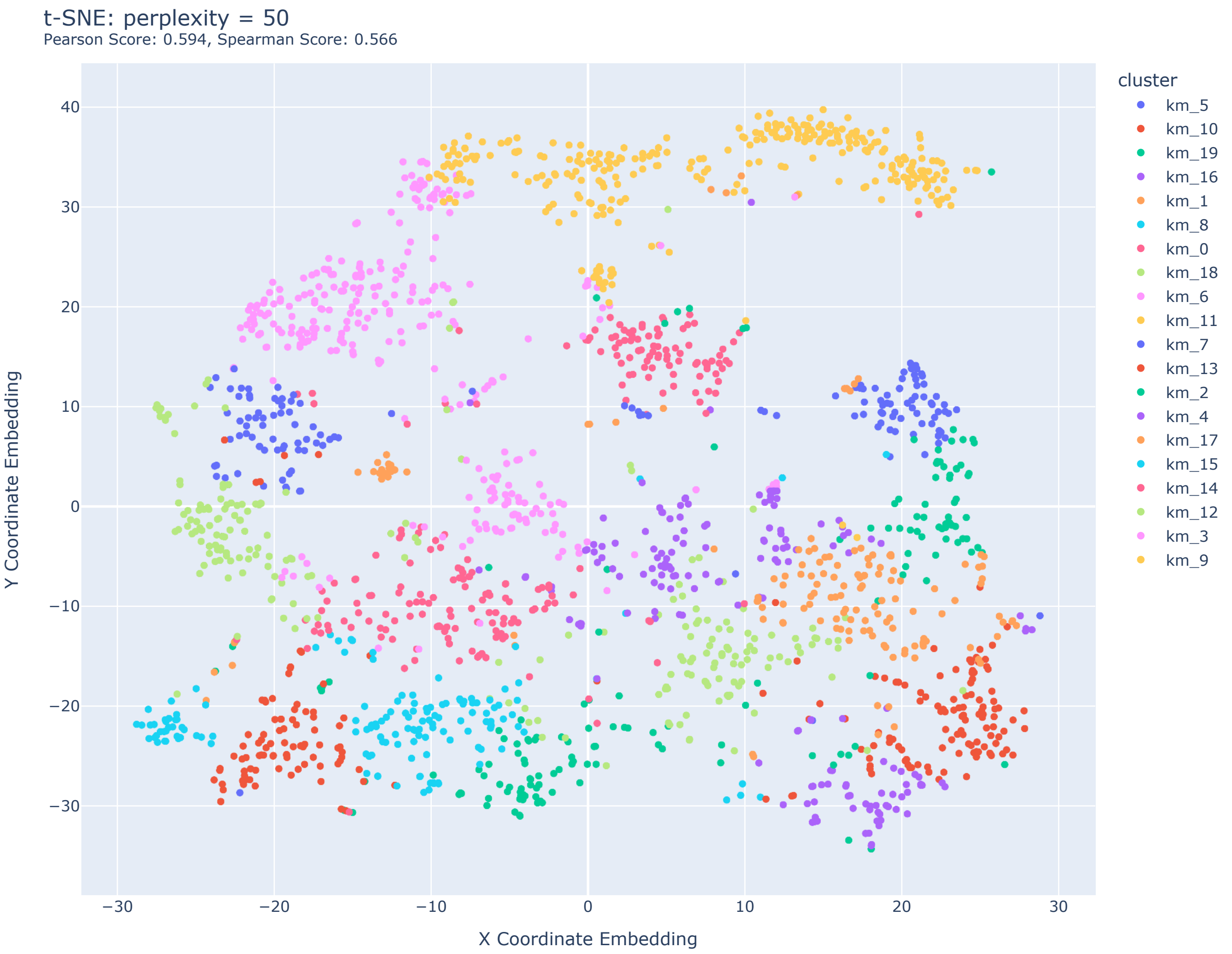

Figure 6: A t-SNE embedding of the ms2deepscore Pleurotus example data. Perplexity was set to 50. The data was subdivided into 20 clusters using k-medoids clustering on the ms2deepscore derived pairwise distance matrix and not on the two-dimensional embedding. Clusters are highlighted in color. Clusters are well separated in the embedding and show only limited overlap with one another. Compared to the run with a perplexity of 30 (figure 4a), slightly increased amounts of overlaps can be seen between clusters.

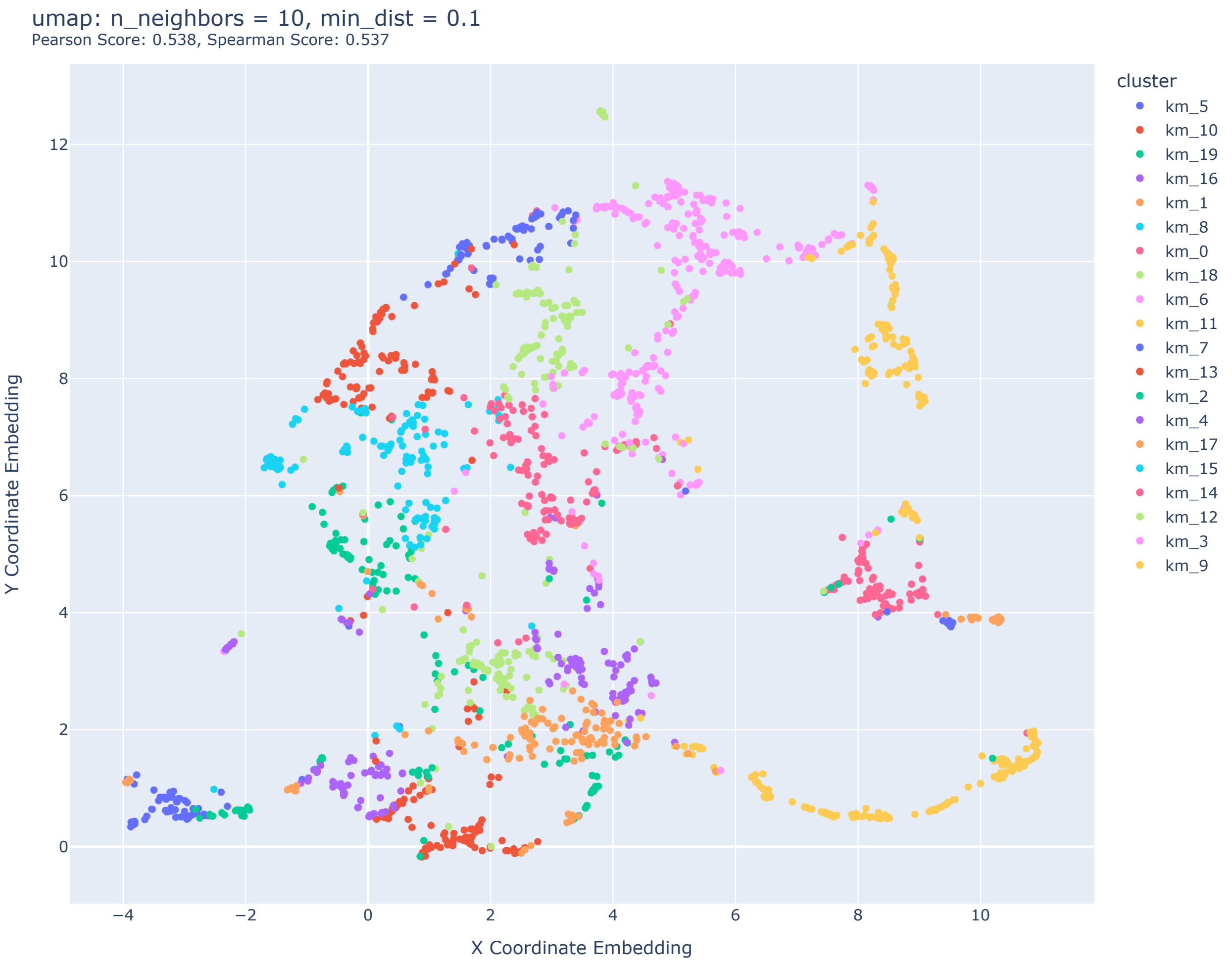

Figure 7: A umap embedding of the ms2deepscore Pleurotus example data. The n-neighbors parameter was set to 10. The data was subdivided into 20 clusters using k-medoids clustering on the ms2deepscore derived pairwise distance matrix and not on the two-dimensional embedding. Clusters are highlighted in color. With low n-neighbor settings many clusters of umap are separable from one another without color. However, many scatter points fall into dense ridges that would make edge overlays difficult to read.

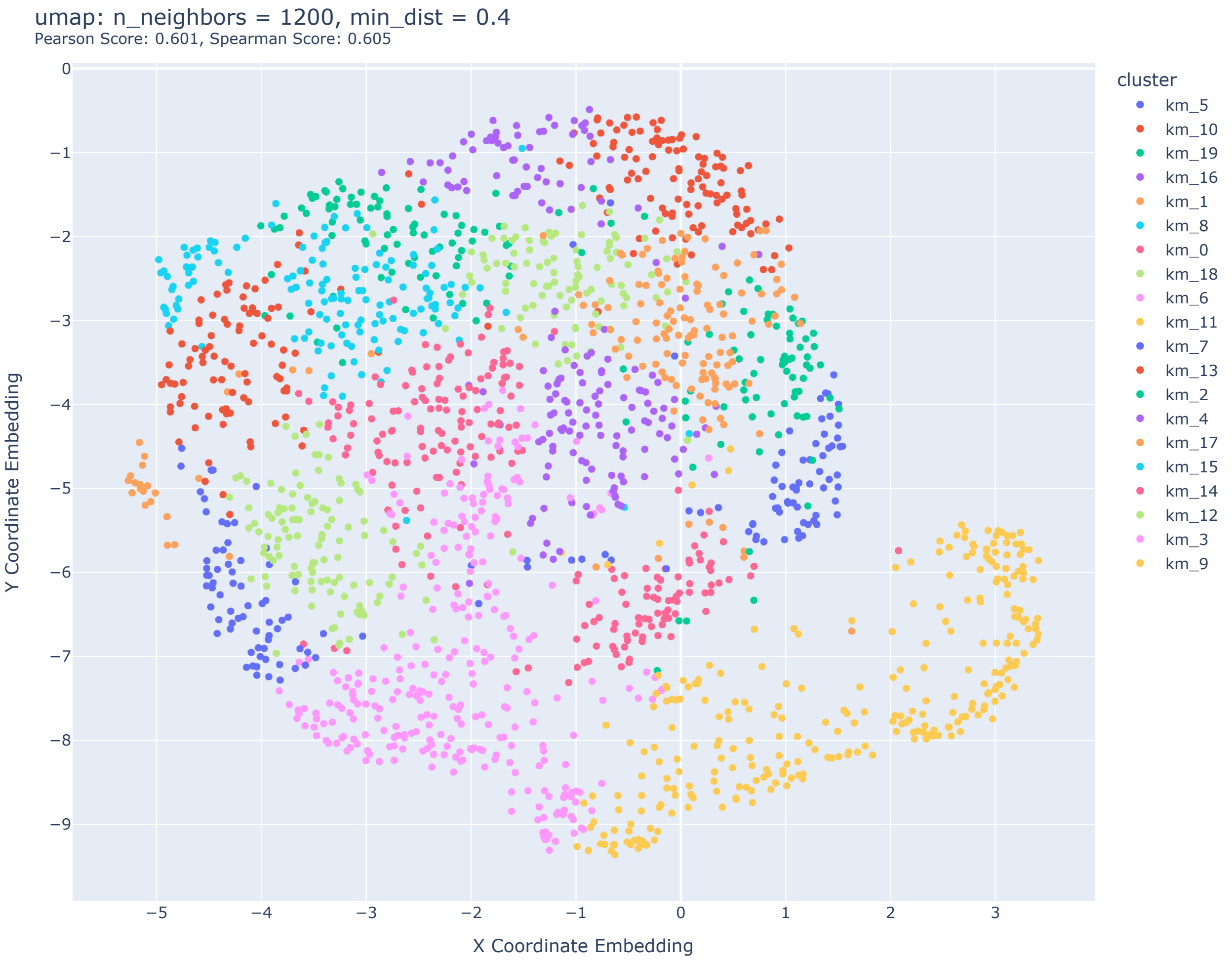

Figure 8: A umap embedding of the ms2deepscore Pleurotus example data. The n-neighbors parameter was set to 1200 and min-dist was set to 0.4 rather than the default of 0.1. The data was subdivided into 20 clusters using k-medoids clustering on the ms2deepscore derived pairwise distance matrix and not on the two-dimensional embedding. Changing the min-dist parameter has set the umap embedding points further apart, reducing the visual distinction of the clusters without color. While the ridges observed for default min-dist are gone, overlap between clusters is aggravated.
